# Isolation and characterization of biofilm-disrupting proteus phage Premi

**DOI:** 10.1038/s41598-025-23545-3

**Published:** 2025-11-13

**Authors:** Guadalupe Valencia-Toxqui, Shobana Sugumar, Jolene Ramsey

**Affiliations:** 1 Department of Biology, Center for Phage Technology, Texas A&M University, College Station, TX USA; 2 Department of Genetic Engineering, Faculty of Engineering and Technology, School of Bioengineering, SRM Institute of Science and Technology, SRM Nagar, Kattankulathur, Kanchipuram, Chennai, Tamil Nadu India

**Keywords:** Proteus, Phage, Multidrug-resistance, CAUTI, Podophage, UTI, Bacteriophages, Biofilms, Phage biology, Microbiology

## Abstract

*Proteus mirabilis* is a biofilm-forming, multidrug-resistant bacterium and one of the most common causes of catheter-associated urinary tract infections (CAUTIs). Phage therapy is an alternative method that can be used to address the problem of multidrug-resistance. In this study, we report isolation and characterization of virulent phage Premi. The phage exhibits lytic activity against 4 out of 30 clinical isolates of *P. mirabilis* tested and is stable when exposed to pH values between 3 and 11. Phage Premi demonstrated significant anti-biofilm activity against *P. mirabilis*, reducing 24-hour established biofilms by 59–68%. In the 42-kb Premi genome, functions were assigned for the 50 predicted protein-coding genes including those involved in DNA replication, DNA modification, and lysis. Structural proteins were verified using mass spectrometry of purified virions. A comparison of its genomic features and phylogenetic analysis revealed that phage Premi is a podophage member of the order *Caudoviricetes* sharing 96% nucleotide similarity with Proteus phage PM 85 and has a T7-like phage genomic organization. Our study shows that Premi effectively inhibits *P. mirabilis* biofilms and could be a promising antimicrobial agent for treating drug-resistant *P. mirabilis* infection.

**Supplementary Information:**

The online version contains supplementary material available at 10.1038/s41598-025-23545-3.

## Introduction

*Proteus mirabilis* are Gram-negative, non-sporulating, facultative anaerobic bacteria belonging to the genus *Proteus* and family *Enterobacteriaceae*, characterized by its remarkable swarming motility and diverse metabolic capabilities^1^. They are frequently found in various environments: soil, water, and the gastrointestinal tracts of animals, including humans^2^. Species within the *Proteus* genus, such as *P. vulgaris*, *P. penneri*, and *P. mirabilis*, are harmful to humans but almost 90% of the infections are caused by *P. mirabilis*^3^. Along with *Escherichia coli*, it is one of the primary causes of catheter-associated urinary tract infections (CAUTI)^4^. The bacterium possesses virulence factors that enable it to adhere to and invade host tissues, contributing to its pathogenicity.

Antibiotic resistance is a growing concern in bacterial infections, and *P. mirabilis* is no exception. Combined with its virulence factors such as flagellar-mediated swarming motility, host cell attachment via fimbriae, and dense biofilm formation on catheters, antibiotic resistance in *P. mirabilis* makes infections difficult to treat^5,6^. The bacterium can develop resistance to various antibiotics through mechanisms such as the production of beta-lactamases and efflux pumps^7^. This resistance poses challenges in the clinical management of infections caused by *P. mirabilis*.

Phage therapy—the study and use of phages in the management of bacterial infections—has rekindled attention as a possible adjunct or substitute for antibiotics. Phages typically infect specific strains or species of bacteria due to their highly specialized host range. This selectivity minimizes the disruption of the helpful microorganisms in the surrounding environment while permitting targeted treatment, which is desirable^8^. *Proteus* phages are being used therapeutically, which is in line with the general interest in investigating phage treatment as a possible remedy for the worldwide problem of antibiotic resistance^9^. *Proteus* phages could prove to be useful weapons in the fight against bacterial illnesses as this field of study develops, providing a more focused and possibly long-lasting form of therapy^10^. Phage cocktails have shown efficacy in preclinical testing for *Proteus* biofilm prevention or reduction^11^. However, as of writing in 2025, only 61 *Proteus* phages have been submitted to GenBank, indicating this group is undersampled from the environment^12^. A larger bank of characterized *Proteus* phages is needed to improve our capability to develop phage therapeutics.

In this study, we isolated a virulent *P. mirabilis* phage we called Premi from the Navasota wastewater treatment plant. We performed TEM analysis for morphology, host range determination, checked parameters such as temperature and pH stability, calculated MOI, and antibiofilm activity of isolated phage Premi was assessed. Additionally, we performed a detailed genomic analysis of Premi. Our characterization of Premi reveals it is a highly effective antibacterial agent against susceptible strains, making it a suitable candidate for inclusion in therapeutic cocktails aimed at tackling the urgent worldwide problem of drug-resistant bacterial infections.

## Results and discussion

### Phage isolation and morphology characterization

**Fig. 1:**
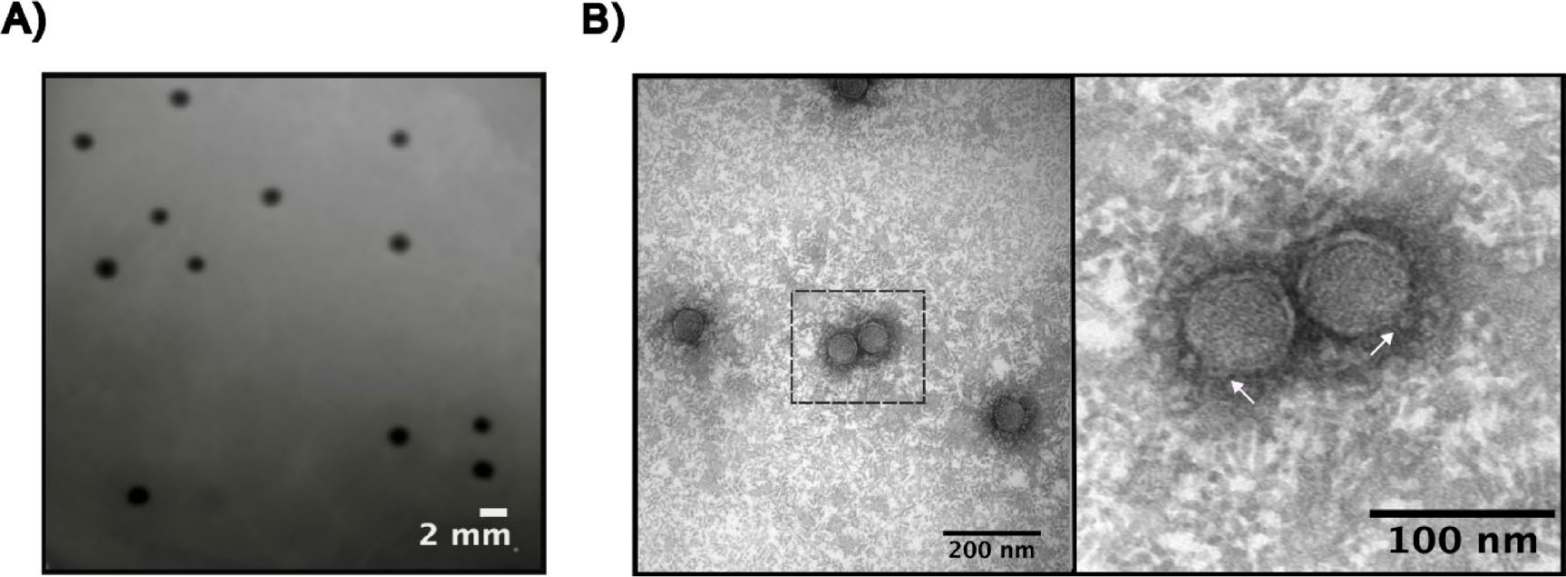
Morphology of phage Premi plaques and virions. A) Premi plaques on the *P. mirabilis* lawn have a large, clear morphology. B) Premi transmission electron micrograph displaying virions with a head diameter ~35 nm. White arrows indicate the location of the short tail.

We isolated the phage Premi from the wastewater treatment plant (sewage influent) in Navasota, Texas, USA with *P. mirabilis* as the host. The plaques were uniform in size, approximately 1 mm in diameter, and exhibited virulent phage characteristics with clear plaques that have sharply defined edges (Fig. 1A). Transmission electron microscopy revealed a head of icosahedral structure with a diameter of approximately ~ 35 nm and a short, non-contractile tail (Fig. 1B). Based on the morphological characteristics of the phage tail, the phage belongs to the class *Caudoviricetes*. The phage was named Proteus phage Premi.

### Phage Premi lysis pattern and host range

**Fig. 2:**
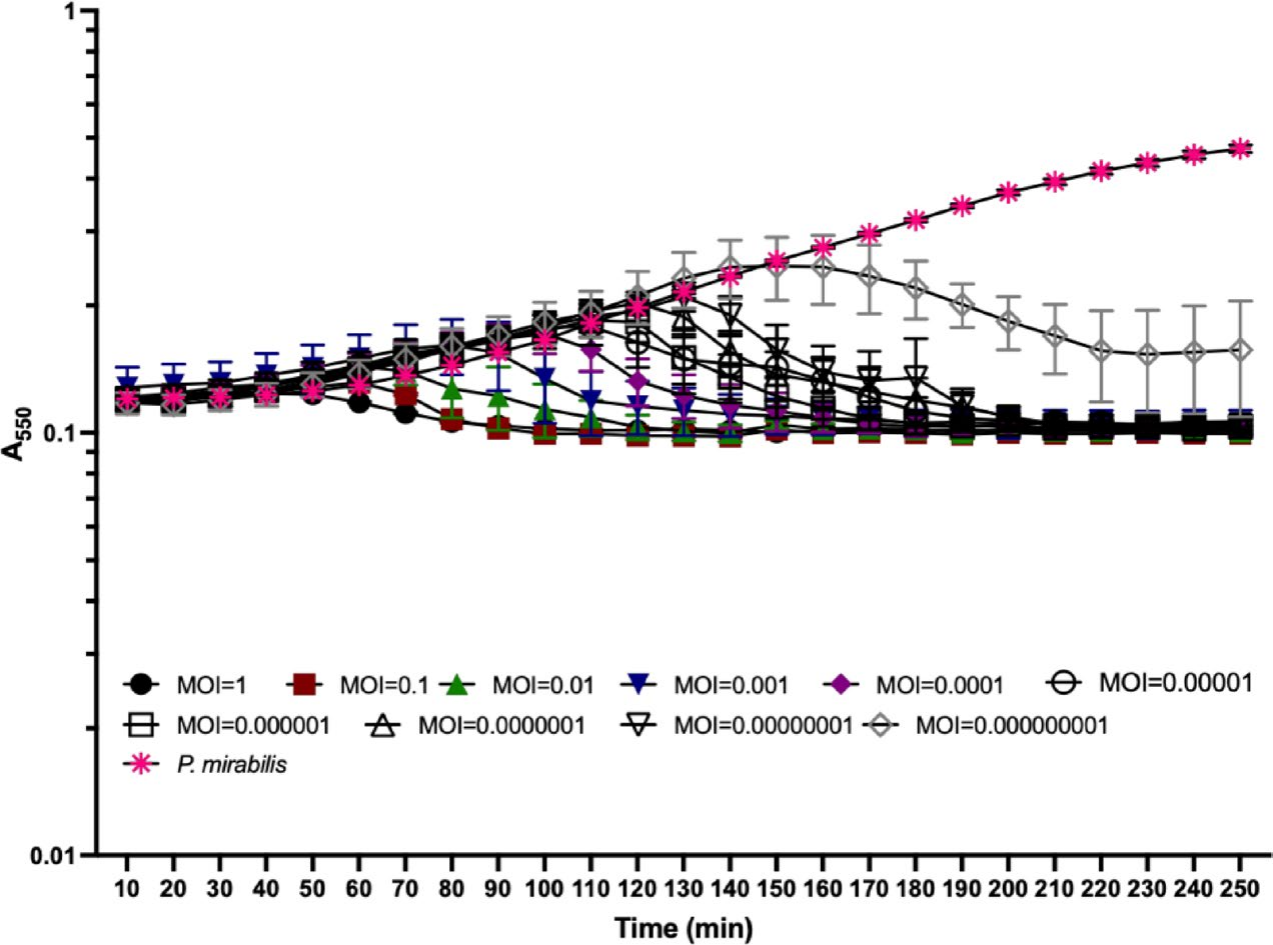
Phage Premi lysis pattern. *P. mirabilis* host cultures were infected at mid-log phase with Premi at a range of MOIs and monitored for host cell lysis. At each time point the mean of two biological replicates with three technical replicates is graphed with one standard deviation shown.

The lysis pattern of phage Premi was investigated over a range of calculated multiplicities of infection (MOI) in liquid culture. Complete population lysis was observed for all infection conditions (Fig. 2), suggesting Premi is a virulent phage.

To investigate the host range of phage Premi, we used a broad panel of bacterial hosts available in the collection at the Center for Phage Technology and clinical isolates gifted from Dr. Subashchandrabose at Texas A&M University. It was found that phage Premi can infect 4 out of 30 strains of *P. mirabilis* tested, including primarily clinical and UTI isolates (Table 1). Overall, the investigation revealed that phage Premi has a narrow range of activity and targets only specific strains of *P. mirabilis*.

### pH stability and inhibition of biofilm formation by phage Premi

**Fig. 3:**
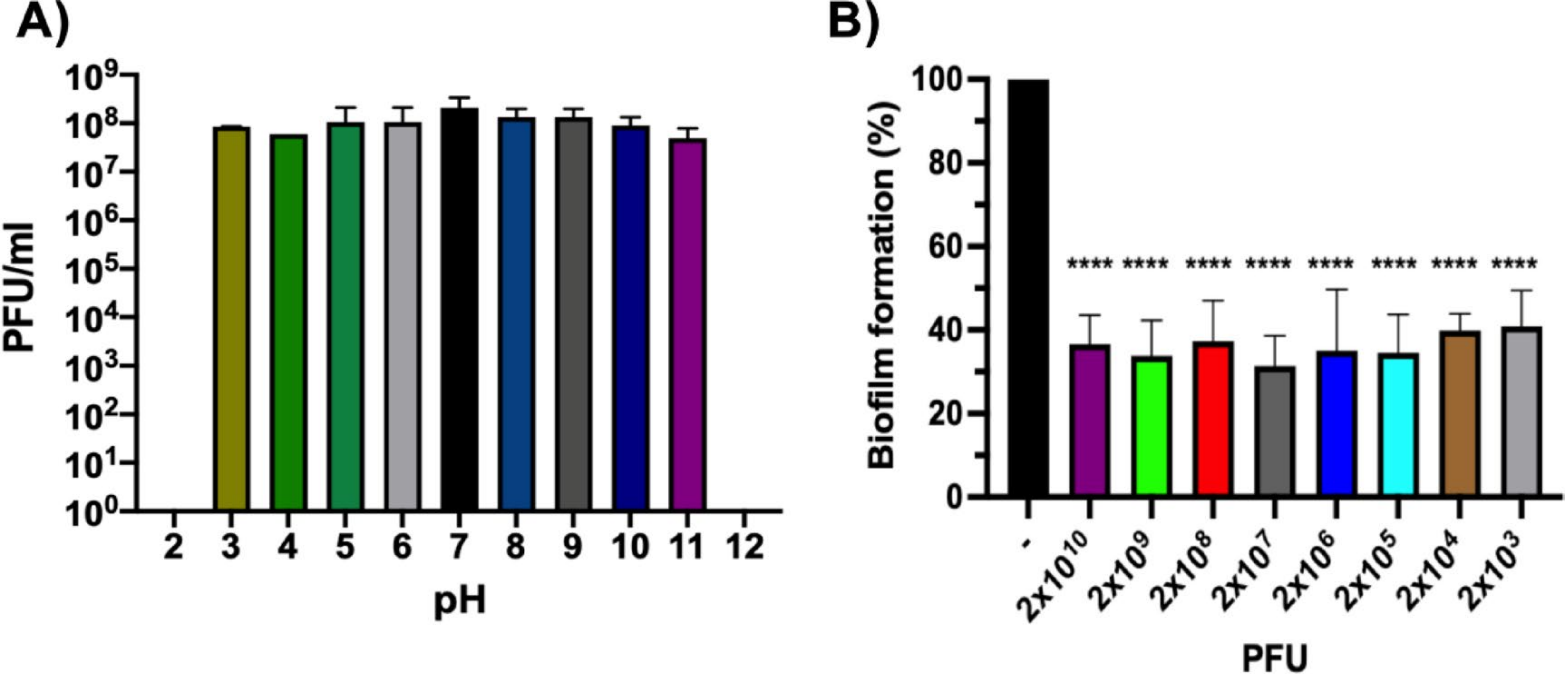
Phage Premi pH stability and anti-biofilm activity. A) Premi stability was measured when exposed to pH 2 to 12 for three hours. B) *P. mirabilis* biofilm formation in plastic dishes as measured by the crystal violet assay after treatment with serial dilutions of phage Premi starting at a concentration of 2x10^10 ^PFU. ANOVA tests with statistically significant reduction compared to the negative control are marked **** for p ≤ 0.0001.

*Proteus* is well-known for forming crystalline biofilms within the urinary tract. It is more tolerant to antibiotics and phages, highlighting Premi’s potential as a tool to control biofilm-mediated bacterial infections. Phage Premi was found to be stable after three hours at pH values between 3 and 11 (Fig. 3). However, it was completely inactivated at pH levels of 2 and 12. To evaluate the biofilm inhibition capability of the phage Premi, established *P. mirabilis* biofilms were treated for 18 h at 37 °C with serial dilutions of phage. The quantification of biofilm mass was accomplished using the crystal violet staining technique. After treatment with Premi, there was a significant reduction in the *P. mirabilis* biofilm biomass in all the dilutions evaluated ranging from 59% to 68% (Fig. 3). These results demonstrated that application of phage Premi effectively reduced the pre-formed biofilms of *P. mirabilis*.

### Genome features and annotation of phage Premi

**Fig. 4:**
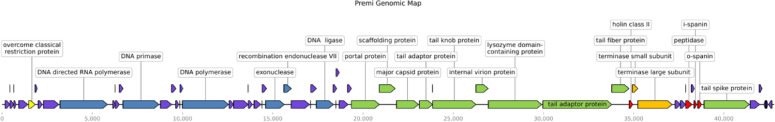
Linear Genome Plot of the annotated Proteus phage Premi genome. The protein-coding genes predicted in the Premi genome with their putative functional assignments are shown. Genes are colored according to arbitrary groupings: defense (yellow), replication (blue), structural (green), packaging (orange), lysis (red), and hypothetical proteins of unknown function (purple).

**Fig. 5:**
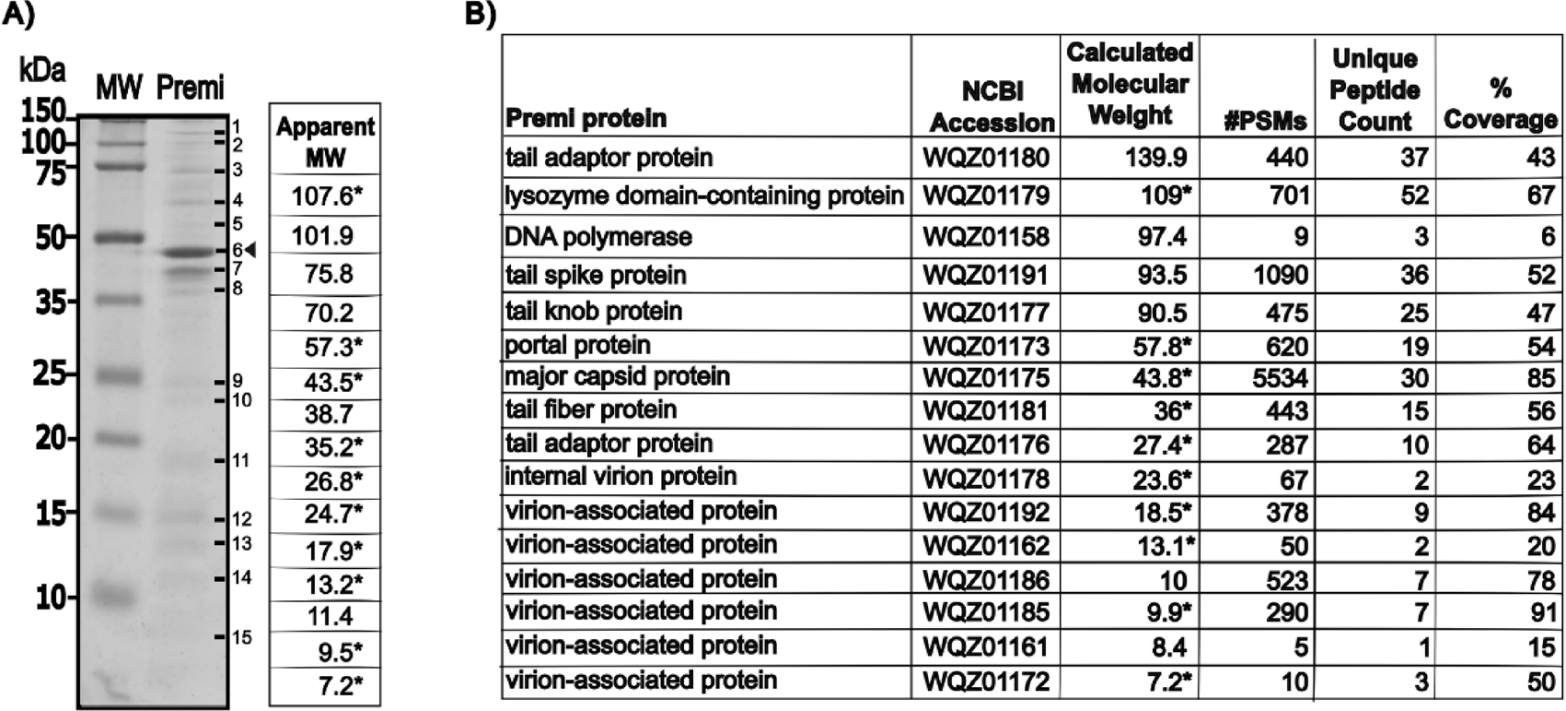
Protein composition of phage Premi virions. A) Phage Premi particles purified by CsCl gradient were separated on a 15% Tris-Tricine SDS-PAGE gel. The arrowhead indicates the position of major capsid protein. An asterisk (*) indicates an apparent molecular weight (MW) that matches that of a protein identified by mass spectrometry. The complete Tris-Tricine SDS-PAGE gel image is shown in Supplementary Figure 1. B) Table of mass spectrometry results for trypsin-digested Premi proteins from whole phage particles. #PSMs is the total number of peptide spectral matches identified for a protein and the unique peptide count is equal to the number of peptide sequences exclusive to the protein.

The complete genome of phage Premi is a 42,785 bp linear dsDNA molecule, with a GC content of 38.7%. Of the 50 genes identified, 23 were assigned predicted functions (Fig. 4). Functional assignments included terminase subunits, portal protein, major capsid protein, tail spike and fiber proteins, internal virion proteins, and replication and transcription proteins. The major capsid protein (43.8 kDa) is observed in SDS-PAGE of highly purified Premi virions (Fig. 5). All the proteins within virions were identified by liquid chromatography tandem mass spectrometry of highly purified phage preparations (Fig. 5). The majority are predicted structural proteins, but some did not have an assigned function.

**Fig. 6:**
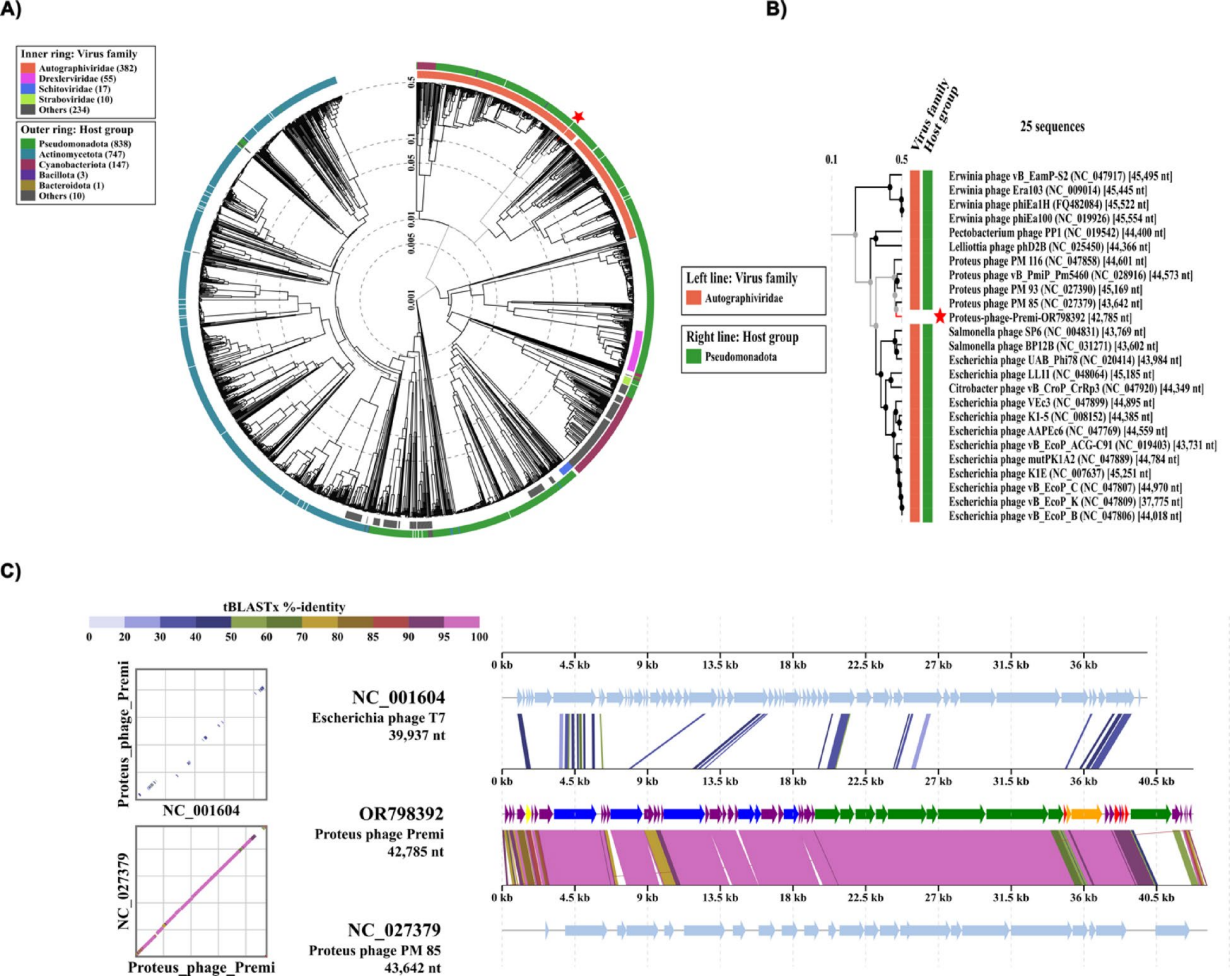
Protein-based phylogenetic analysis of phage Premi. A) A dendrogram representing the global genomic comparison of predicted proteins from phage Premi with other viral sequences by tBLASTx using VipTree. The colored rings surrounding the dendrogram represent current ICTV virus families (inner ring) and host groups (outer ring). The red star represents the position where phage Premi is located. B) An excerpt of the proteomic tree showing phage Premi and the 24 viruses that are most closely classified. C) At left, pairwise whole genome dot plots. At right, predicted proteins and identity detected by tBLASTx between Proteus phages Premi (OR798392) and PM 85 (NC_027379) or Escherichia phage T7 (NC_001604) in a syntenic alignment. Analysis performed using VipTree Proteomic Tree online server.

The complete phage genome sequence was compared using the BLASTn (megablast) webserver. Among the hits obtained, phage Premi has 96% nucleotide identity over 90% of the genome with Proteus phage PM 85^10,13^ and similar phages Proteus phage PM 116 (NCBI Acc. NC_047858.1), Pm93 (NCBI Acc. NC_027390.1), Pm5460 (NCBI Acc. NC_028916.1), BigMira-UFV-01 (NCBI Acc. OQ729741.1), and MidiMira-UFV02 (NCBI Acc. OQ729742.1). Based on these similarities, phage Premi is a species within the *Acadevirus* classification. Additional protein-based comparisons performed with VipTree v4.0 revealed distant relationships with many other T7-like podophages (Fig. 6).

## Conclusions

Premi is a newly discovered phage that infects *Proteus* species, a host of significant concern for its contributions to antibiotic-resistant bacterial infections in humans. Characterization of Premi reveals that it has a typical podovirus morphology and virulent infectious cycle. Premi exhibits stability and infectivity at a wide range of pH values (3 to 11), which may make it suitable for phage therapy specifically targeting *P. mirabilis* in the urinary tract. Its fully sequenced and analyzed genome shows a T7-like organization for podoviruses with structural and enzymatic genes necessary for replication and host cell lysis. Genomic comparison with other phages reveals that it belongs to the class *Caudoviricetes*, family *Autographiviridae*, and appears to be devoid of integrase genes, antibiotic-resistance genes, virulence factors, and toxin genes. Premi demonstrated lytic activity against three clinical isolates beyond its isolation host out of 30 hosts tested. This apparent narrow host specificity for Premi and other *Proteus* phages requires further investigation in a host panel which is well-characterized for its diversity and complexity^10,13–15^. Given the rising concerns over antibiotic resistance in *Proteus* infections, and the limited characterization of *Proteus*-infecting phages worldwide (only 61 total present in the public NCBI database), future studies with Premi may augment ongoing work to develop preventatives and treatments for CAUTIs^8,9,11^. As has been the case for other human pathogens, it is likely effective phage therapy against *Proteus* will require multi-phage cocktails for full efficacy^11^. Further research into Premi should explore its interaction dynamics with a wider range of well-described *Proteus* and other Gram-negative species and optimize its production and purification processes for potential therapeutic use. Ultimately, Premi exemplifies the ongoing discovery and characterization of phages contributing to the growing arsenal of tools for combating bacterial pathogens and advancing our understanding of viral diversity in natural environments.

## Methods

### Bacterial strains and growth conditions

*P. mirabilis* (ATCC 29906) were cultured in Nutrient Broth at 30 °C. Clinical strains of *P. mirabilis*, a kind gift of Sargurunathan Subashchandrabose at Texas A&M University, were cultured in Lysogeny Broth (LB) medium at 37 °C.

### Phage isolation and purification

*P. mirabilis* were cultured in 10 mL NB broth and shaken for 12 h at 30 °C, 150 rpm until the logarithmic phase. The enrichment process involved combining 100 µl of bacterial culture, 40 mL of filtered sewage, and 10 mL of NB broth samples, and incubating the mixture in an incubator shaker at 26 °C for 24 h. The resulting phage-enriched mixture was centrifuged at 8000 *xg* for 10 min and the supernatant was transferred to a fresh tube. To 1 mL of the filtrate, 20 µl of chloroform was added and clarified at top speed for 1 min. The double-layer agar method was used to determine the presence and of the phage, with some modifications. A mixture of 100 µl of bacterial cells (~ 1 × 10^8^ CFU/mL) and 50 µL of the filtrate sample was added to NB soft agar medium (0.4% agar). The mixture was then spread onto the NB agar medium, air-dried, and incubated at 37 °C for 4–5 h. The resulting plaques were visualized, and a single plaque was picked up using a sterilized tip and resuspended in lambda dilution buffer (100 mM NaCl, 50 mM Tris-HCl pH 7.6, 8 mM MgSO_4_, 1% gelatin).The plaque purification was repeated three times until consistent size and transparency were achieved. The purified phage was kept at 4 °C for subsequent experimental procedures. To the high titer phage lysate, 10% wt/vol PEG-8000 Premi was added and kept overnight to concentrate and purify, as previously described^16^. The phages were further resuspended in SM buffer (100 mM NaCl, 8 mM MgSO_4_⋅7H_2_O, and 50 mM Tris-HCl pH 7.5), and CHCl_3_ was added to remove the PEG-8000 from the phages.

### Transmission electron microscopy (TEM)

After purification of phage Premi by CsCl gradients (1.7, 1.5, 1.3 g/mL), phage particles were deposited on a Formvar carbon-coated copper grid and negatively stained with 2% uranyl acetate. Images were collected at the Microscopy and Imaging Center at Texas A&M University using a JEOL 1200 EX TEM at a 100-kV accelerating voltage.

### Phage amplification

The phage was propagated by the double-agar overlay method using overnight saturated *P. mirabilis* host bacterial culture. For liquid lysate amplification, the host was subcultured and grown to a mid-exponential OD_550_ of 0.4 before infection at various multiplicities of infection. For quantification of phage yield, plates with a plaque count ranging from 30 to 300 were selected for the enumeration of phage titers reported in plaque-forming units/milliliter (PFU/mL). Three biological replicates were used to select the infection conditions yielding maximum phage amplification.

### Effect of pH on phage stability

To test pH stability, the lambda dilution buffer was adjusted to the pH values (2, 3, 4, 5, 6, 7, 8, 9, 10, 11, 12) and the phage (~ 10^9^ PFU/mL) was then added and incubated at 37 °C for 3 h. The phage titer was determined by the double-layer agar method. Each sample was assayed in triplicate^17^.

### Genome sequencing and annotation

The DNA of the phage was extracted using the phage DNA Isolation Kit from Norgen Biotek Corp., Canada. This was done after the PEG precipitation of crude lysate. Samples were prepared for Illumina sequencing at SeqCoast Genomics (Portsmouth, NH, USA) with an Illumina DNA Prep tagmentation kit and unique dual indexes. Sequencing was performed on the Illumina NextSeq2000 platform using a 300 cycle flow cell kit to produce 2 × 150 bp paired reads. The 396,576 sequence reads from the index containing the phage genome were quality-controlled with FastQC (https://www.bioinformatics.babraham.ac.uk/projects/fastqc/). The phage genome was then assembled into a single raw contig using SPAdes v.3.5.0 with 636.7-fold coverage after trimming with the FASTX-Toolkit 0.0.14^18^. Finally, PCR amplification across the raw contig ends was done with a forward primer 5′-aaccggcttacctgctgcaacc-3′ and a reverse primer 5′-ttgtgggcgctggctgtaatgg-3′. Sanger sequencing of the DNA product verified that the contig sequence was complete. The Premi genome was reopened at short direct terminal repeats of 473 bp predicted by PhageTerm, as expected for a T7-like phage.

### Host range determination

The host range of purified phage Premi was determined using efficiency of plaquing (EOP) with different clinical *P. mirabilis* isolates. The study also included other Gram-negative bacteria, comprising various clinical isolates, as shown in Table 1^19,20^. A culture lawn was prepared on LB agar medium. After 30 min of incubation, 10 µL of serial dilutions of Premi lysate was spotted onto the lawn surface. The EOP was calculated by dividing the viral titer of each strain tested by the viral titer of the isolation host strain (ATCC 29906). The experiment utilized a collection of *P. mirabilis* strains, including both ATCC and clinical strains, as well as other bacteria belonging to the *Enterobacteriaceae* family and Gram-positive bacteria listed in Table 1.

### Antibiofilm activity of phage Premi

The *P. mirabilis* was examined for their ability for biofilm formation using the microtiter plate assay method. Biofilm formation was quantified using a 96-well flat-bottomed polystyrene microtiter plate. Briefly, an OD_550_ = 0.2 culture of *P. mirabilis* incubated at 37 °C in lysogeny broth was prepared for the assay. After that, 200 µl of bacterial suspensions were added to each well, and the plate was incubated at 37 °C for 24 h. The pre-formed biofilms were washed three times with sterile tap water. The wells were emptied, dried, and stained with 200 µl of 0.1% crystal violet solution for 5 min. The stain was rinsed off, and the wells were washed under running tap water. The wells were then air dried for 30 min prior to solubilization with 200 µl of 90% (volume/volume) Ethanol. This assay was conducted in triplicate. Optical density (OD) was measured at 595 nm with a plate reader. All measurements were expressed as the mean plus or minus one standard deviation. To evaluate phage efficiency against established biofilm, serial dilutions of filtered phage were prepared in lambda dilution buffer (10 mM Tris-HCl, pH 7.2, 10 mM MgCl_2_, and 0.1% gelatin). For each treatment 150 µl of fresh LB plus 50 µl of each phage dilution were added to the established biofilms produced on the microtiter plates by *P. mirabilis*. One set of negative control wells contained 200 µl of lysogeny broth alone without bacterial culture, and another contained established bacterial biofilms inoculated with 200 µl of lysogeny broth and 50 µl of lambda dilution buffer. The plates were incubated at 37 °C for 18 h to assess the time-dose effect of phage application on the established biofilms. The remaining biofilm biomass was stained and quantified as previously described to determine the final biofilm density. Triplicate measurements were expressed as the mean plus or minus one standard deviation. Statistical significance of differences in biofilm reduction was assessed by one-way ANOVA, followed by Sidak’s multiple comparation test.

### Genome annotation and bioinformatic analysis

The Premi complete contig was assembled, analyzed, and annotated in the Center for Phage Technology Galaxy and Web Apollo interfaces (https://cpt.tamu.edu/galaxy-pub*)*, as described by Ramsey et al. (2020)^21^. Structural annotation was performed using GLIMMER v3^22^ and MetaGeneAnnotator v1.0^23^, while tRNA coding regions were predicted by ARAGORN v2.36^24^. The function of the called genes was predicted by BLAST v2.9.0^25^ against the NCBI non redundant (nr) and Swiss-Prot databases, InterProScan v5.33^26^, and TMHMM v2.0^27^, progressiveMauve v2.4^28^ was used to calculate the genome-wide DNA sequence similarity. Phage termini were predicted using PhageTerm^29^. All software was run at default settings. All BLAST queries were run against the NCBI nonredundant and UniProtKB Swiss-Prot and TrEMBL databases^30,31^. Supporting analysis was performed using the HHSuite v3.0 HHpred tool (multiple sequence alignment generation with the HHblits ummiclus30_2018_08 database and modeling with PDB_mmCIF70)^32^. The genome sequence and associated data for phage Premi were deposited under GenBank accession no. OR798392, BioProject accession no. PRJNA222858, Sequence Read Archive accession no. SRR26984153 and BioSample accession no. SAMN38498658. The genome map was prepared using the Genome Linear Plot tool in CPT Galaxy, modified from DNA features viewer^21,33^. Global genome comparison at the proteome level was performed by tBLASTx using the ViPTree 4.0 server on default settings^34^.

### Analysis of phage structural proteins by mass spectrometry

A 20 µg sample of whole Premi particles from a CsCl-purified stock was prepared for mass spectrometry analysis as previously described^35^. Virion proteins were separated by SDS-PAGE on a 16% Tris-glycine gel with ~ 10^8^ PFU per lane, visualized with Coomassie blue, and compared to the Precision Plus Protein™ Dual Xtra Prestained Protein standard (Bio-Rad). Briefly, after methanol-chloroform precipitation, reduction and alkylation with on-column trypsin digestion, the samples were analyzed by LC-MS/MS using an Ultimate 3000 nano-LC system coupled to an Orbitrap Fusion Lumos Tribrid mass spectrometer (Thermo Scientific).

**Table 1:** Host range analysis of Premi. Quantitative Lysis tests were performed in triplicate. The efficiency of plaquing was calculated by dividing the phage titer on each tested strain by the phage titer on the isolation host. Sources: 1-Center for phage technology (CPT) Texas A&M University; 2-gift from Subashchandrabose lab at Texas A&M University; 3-from K. Frank, NIH, gift to CPT; 4-from GangaGen, gift to CPT.

Host organism	Strain		Source	Efficiency of Plaquing
*Proteus mirabilis*	ATCC 29,906	Isolation host	1	1.0
*Proteus mirabilis*	M6, #1	Clinical isolate	3	2.2 × 10^−7^± 3.1 × 10^−8^
*Proteus mirabilis*	M6, #2	Clinical isolate	3	2.9 ± 0.3
*Proteus mirabilis*	M6, #6	Clinical isolate	3	1.9 ± 0.3
*Proteus mirabilis*	ATCC 25,933	Clinical isolate	1	0.0
*Proteus mirabilis*	ATCC 7002	Clinical isolate	1	0.0
*Proteus mirabilis*	ATCC 43,071	Clinical isolate	1	0.0
*Proteus mirabilis*	ATCC 35,659	Quality control strain	1	0.0
*Proteus mirabilis*	M6, #3	Clinical isolate	3	0.0
*Proteus mirabilis*	M6, #4	Clinical isolate	3	0.0
*Proteus mirabilis*	M6, #5	Clinical isolate	3	0.0
*Proteus mirabilis*	M6, #7	Clinical isolate	3	0.0
*Proteus mirabilis*	M6, #8	Clinical isolate	3	0.0
*Proteus mirabilis*	M6, #9	Clinical isolate	3	0.0
*Proteus mirabilis*	M5, #10	Clinical isolate	3	0.0
*Proteus mirabilis*	HI4320	UTI isolate	2	0.0
*Proteus mirabilis*	WF10	UTI isolate	2	0.0
*Proteus mirabilis*	WF15	UTI isolate	2	0.0
*Proteus mirabilis*	WF32	UTI isolate	2	0.0
*Proteus mirabilis*	WF44	UTI isolate	2	0.0
*Proteus mirabilis*	WF56	UTI isolate	2	0.0
*Proteus mirabilis*	WF57	UTI isolate	2	0.0
*Proteus mirabilis*	WF64	UTI isolate	2	0.0
*Proteus mirabilis*	WF67	UTI isolate	2	0.0
*Proteus mirabilis*	WF75	UTI isolate	2	0.0
*Proteus mirabilis*	WF78	UTI isolate	2	0.0
*Proteus mirabilis*	WF81	UTI isolate	2	0.0
*Proteus mirabilis*	WF89	UTI isolate	2	0.0
*Proteus mirabilis*	WF99	UTI isolate	2	0.0
*Proteus mirabilis*	WF113	UTI isolate	2	0.0
*Proteus mirabilis*	B446	Unknown	4	0.0
*Acinetobacter baylyi*	ADP1	Unknown	1	0.0
*Acinetobacter radioresistens*	HM-107 (SK82)	BEI resources	1	0.0
*Citrobacter freundii* complex	M5, #3	Unknown	3	0.0
*Citrobacter koseri*	M5, #2	Unknown	3	0.0
*Enterobacter aerogenes*	M6, #3	Unknown	3	0.0
*Enterobacter cloacae* complex	M5, #1	Unknown	3	0.0
*Enterobacter cloacae* complex	M6, #2	Unknown	3	0.0
*Enterobacter pasteurii*	ATCC 23,355	Unknown	1	0.0
*Enterococcus faecalis*	ATCC 29,212	Clinical isolate	1	0.0
*Enterococcus faecium*	HM-204 (TX1330)	BEI resources	1	0.0
*Escherichia coli*	ATCC 25,922	Clinical isolate	1	0.0
*Klebsiella pneumoniae*	KPC 27 E3(r) 1	ST258 cps 2 derivative	1	0.0
*Klebsiella pneumoniae*	KPC 27 D2(r) 1	ST258 cps 2 derivative	1	0.0
*Klebsiella pneumoniae*	KPC 27 Soft (r) 1	ST258 cps 2 derivative	1	0.0
*Klebsiella pneumoniae*	1760c Pharr/JR(r)	1760c derivative	1	0.0
*Klebsiella quasipneumoniae*	ATCC 700,603	Clinical isolate	1	0.0
*Morganella morganii*	M5, #1	Unknown	3	0.0
*Morganella morganii*	M5, #2	Unknown	3	0.0
*Morganella morganii*	M5, #3	Unknown	3	0.0
*Plesiomonas shigelloides*	M5, #4	Unknown	3	0.0
*Pseudomonas aeruginosa*	PAO1	Lab stock	1	0.0
*Pseudomonas aeruginosa*	ATCC 27,853	Clinical isolate	1	0.0
*Serratia marcescens*	M5, #2	Unknown	3	0.0
*Serratia ureilytica*	M5, #3	Unknown	3	0.0
*Staphylococcus aureus*	ATCC 29,213	Clinical isolate	1	0.0

## Supplementary Information

Below is the link to the electronic supplementary material.

Supplementary Material 1

Supplementary Material 2

## Data Availability

The genome sequence and associated data for phage Premi were deposited under NCBI GenBank accession no. OR798392, BioProject accession no. PRJNA222858, Sequence Read Archive accession no. SRR26984153 and BioSample accession no. SAMN38498658.
