# Supplementary material for: Isolation and characterization of biofilm-disrupting proteus phage Premi
Source: Sci Rep. 2025 Nov 13;15:39780. doi: 10.1038/s41598-025-23545-3 (PMC12615708; doi:10.1038/s41598-025-23545-3)
Supplement: Supplementary file 1 — Supplementary Material 1 [file 41598_2025_23545_MOESM1_ESM.docx]

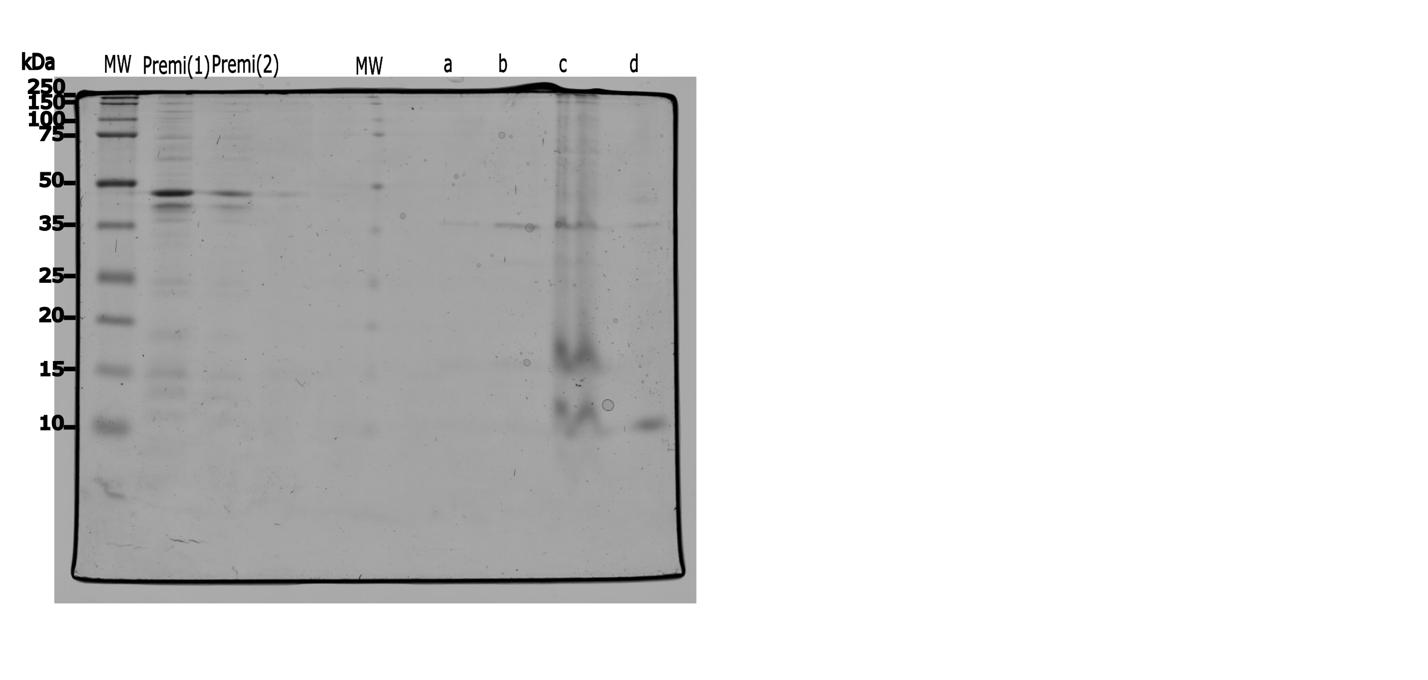
 **Supplementary Figure 1. Complete SDS-PAGE of Premi proteins.**

Premi (1) and Premi (2) samples were boiled and run on a 15% Tris-Tricine SDS-PAGE gel. Wells a–d contained samples unrelated to this project. The image was captured using the LI-COR Odyssey M (v1.2.0.72) in the 630 channel for Coomassie stain, color gray.
