# Supplementary figures and images for: Isolation and characterization of biofilm-disrupting proteus phage Premi
Source: Sci Rep. 2025 Nov 13;15:39780. doi: 10.1038/s41598-025-23545-3 (PMC12615708; doi:10.1038/s41598-025-23545-3)

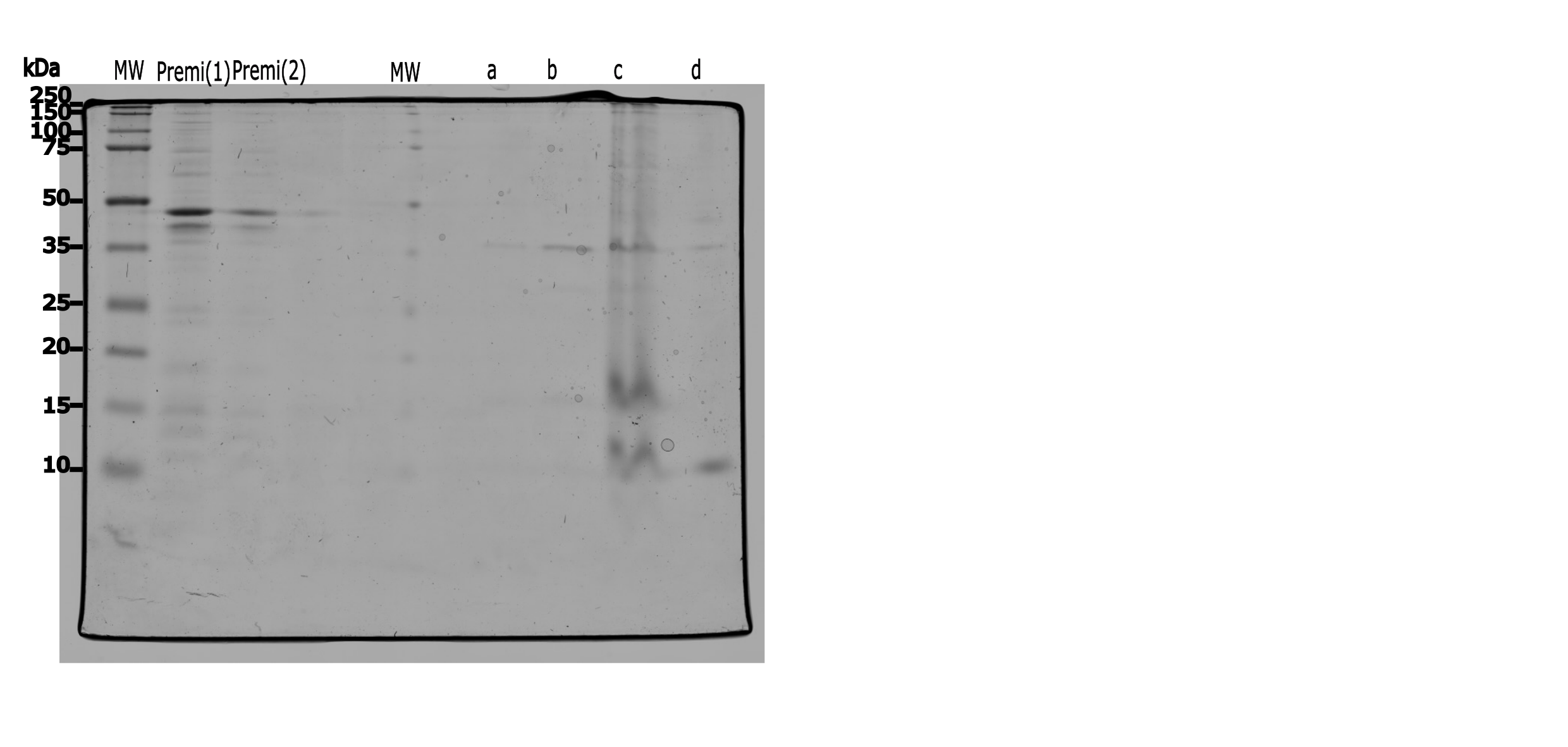

Supplement: Supplementary file 2 — Supplementary Material 2 [file 41598_2025_23545_MOESM2_ESM.tiff]
